# The Features of Extrahepatic Collateral Arteries Related to Hepatic Artery Occlusion and Benefits in the Transarterial Management of Liver Tumors

**DOI:** 10.1155/2013/535272

**Published:** 2013-10-01

**Authors:** Lin Yang, Xiao Ming Zhang, Yong Jun Ren, Nan Dong Miao, Xiao Hua Huang, Guo Li Dong

**Affiliations:** Sichuan Key Laboratory of Medical Imaging, Department of Radiology, Affiliated Hospital of North Sichuan Medical College, Nanchong, Sichuan 637000, China

## Abstract

*Purpose*. To investigate the extrahepatic collateral arteries related to hepatic artery occlusion (HAO) and to determine its benefits in the transarterial management of liver tumors. *Methods and Findings*. Eleven patients (7 hepatocellular carcinomas, 3 liver metastases, and 1 with hemangioma) with HAO confirmed with digital subtraction angiography (DSA) were admitted to our hospital. Of the 11 patients, 7 were men and 4 were women, with an average age of 41.5 ± 15.5 years (range: 29 to 70 years). DSA was performed to evaluate the collateral routes to the liver. In the 11 patients with HAO, DSA showed complete occlusion of the common hepatic artery in 9 patients and the proper hepatic artery (PHA) in 2 patients. Extrahepatic collateral arteries supplying the liver were readily evident. The collateral arteries originated from the superior mesenteric artery (SMA) in 8 patients, from the gastroduodenal artery in 2 patients, and from the left gastric artery (LGA) in 1 patient. Transcatheter treatment was successfully performed via the collateral artery in all patients except the one who had hemangioma. *Conclusions*. DSA is an effective method for detecting collateral circulation related to HAO and may provide information to guide transcatheter management decisions.

## 1. Introduction

Transarterial chemoembolization and infusion chemotherapies have been widely used to treat liver neoplasms [1–5]. However, certain complications following transarterial treatment, such as femoral nerve injury, liver cancer rupture, duodenum perforation, liver abscesses, and hepatic artery occlusion (HAO), were sometimes reported [6, 7]. Above all, HAO is a serious complication because it prevents continuation of transarterial therapy.

It was previously reported that the collateral arterial supply to the liver was evident immediately after HAO [8–10]. It is essential to study the collateral circulation related to HAO in transcatheterization procedures for liver tumor management [11]. Previous reports have indicated that computed tomography (CT) and computed tomography angiography (CTA) could reveal the arterial collateral system in patients with HAO [12, 13]; however, to the best of our knowledge, few studies have addressed the features of extrahepatic collateral routes related to HAO by using digital subtraction angiography (DSA) and benefits in the transarterial management of liver tumors.

In our practice, we primarily found that the collateral pathways after HAO were different from those reported in the literature [12, 13], and the details of the extrahepatic collateral routes to the liver in patients with HAO based on using DSA are unclear. We therefore conducted this study to investigate the extrahepatic collateral routes related to HAO and to determine its benefits in the transarterial management of liver tumors.

## 2. Methods

### 2.1. Objectives

We conducted this study to investigate anatomical changes to the extrahepatic collateral arteries related to HAO based on DSA and to determine the benefits of this method in the transarterial management of liver tumors. 

### 2.2. Participants

From November, 2003 to May, 2011, a total of 431 transarterial procedures were performed in 348 patients with liver tumors at our hospital. The patients with complete occlusion of the common hepatic artery (CHA) or the proper hepatic artery (PHA) related to interventional procedures were included in this study. A total of 11 cases (7 hepatocellular carcinomas, 3 liver metastases, and 1 hemangioma) with HAO were retrospectively studied. Of the 11 patients, 7 were men and 4 were women, with an average age of 41.5 ± 15.5 years (range: 29 to 70 years). 

### 2.3. Angiography and Transcatheterization Procedures

Two radiologists, one with more than 20 years of experience and the other with 8 years of experience in interventional radiology, performed the angiography and transcatheterization procedures. Angiography was performed in a DSA unit (LCV plus, GE Medical Systems) with the use of a 5-F RH angiographic catheter (Terumo, Japan) in all eleven patients to study the collateral arteries related to HAO, to assess the location and vessels feeding the tumors, and to determine the optimal catheter position for chemoembolization or infusion chemotherapy. Sometimes, a 3-F SP microcatheter (Terumo, Japan) was also used to perform the catheterization. The celiac axis and SMA were evaluated in all the patients, and the LGA was evaluated in onepatient. In all the patients except one patient with hemangiomas, DSA was followed by hepatic arterial infusion chemotherapy or chemoembolization (the patient with hemangiomas was scheduled to undergo liver resection). For the hepatic arterial infusion chemotherapy, 5-fluorouracil (1000 mg to 1500 mg), hydroxycamptothecin (30 mg to 40 mg), and adriamycin (40 mg to 50 mg) were administered. Chemoembolization was performed with the administration of the antitumor drugs mentioned above, followed by lipiodol. The volume of the embolus was determined by considering the hepatic serum functional indices (serum albumin level, serum bilirubin level, and prothrombin time ratio) and the diameter of the lesion. The chemoembolization procedure was stopped when the tumor stain disappeared or decreased or when the patient could no longer tolerate the procedure.

### 2.4. Ethics

This study was approved by our institutional review board, and patient informed consent (written consent) was obtained.

## 3. Results

**Figure 1:**
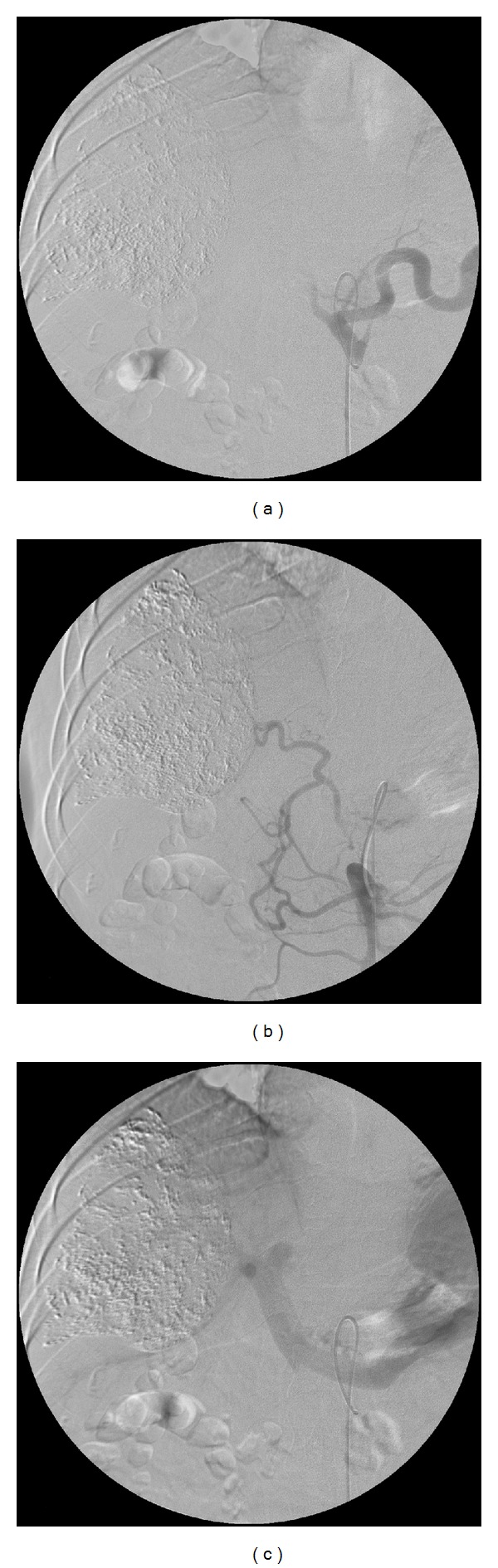
A 46-year-old man with hepatocellular carcinoma. (a) Four weeks after chemoembolization, digital subtraction angiography via the celiac axis showed complete occlusion of the common hepatic artery. (b) Angiography demonstrated the collateral arteries that originated from the superior mesenteric artery. (c) Angiography showed good opacification of the portal vein.

**Figure 2:**
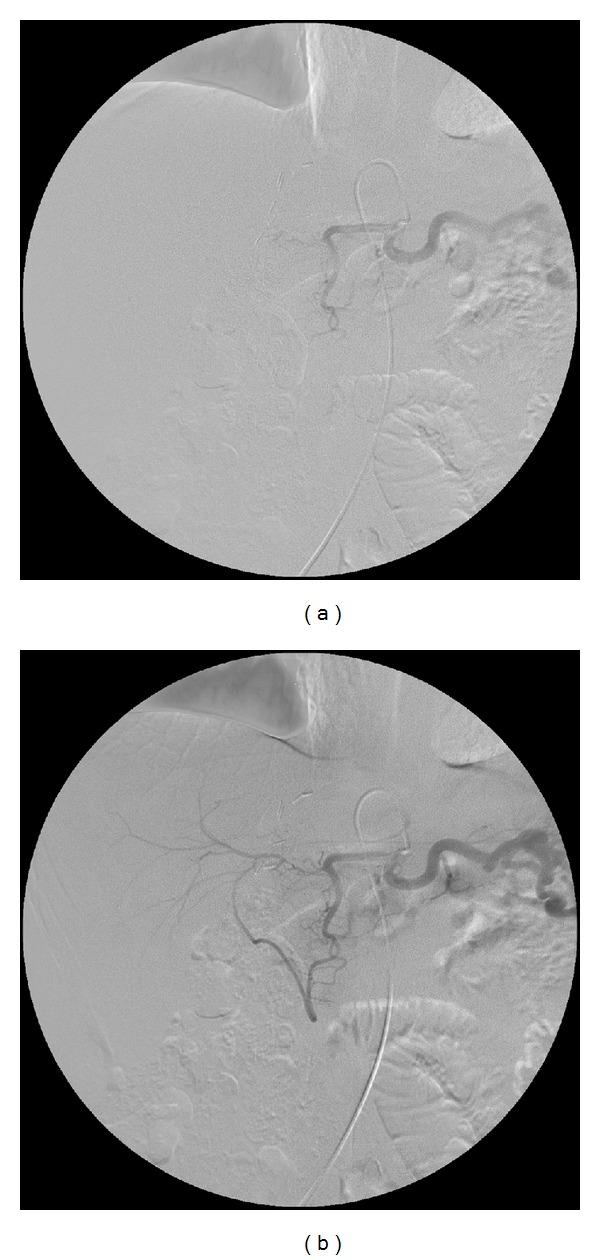
A 70-year-old man with hepatocellular carcinoma. (a) Angiography via the celiac axis showed complete occlusion of the proper hepatic artery. (b) Angiography demonstrated the collateral arteries originated from the gastroduodenal artery.

In all eleven patients, there were good ratings of DSA acquisition. DSA showed complete occlusion of the CHA in 9 patients (81.8%) and the PHA in 2 patients (18.2%). Extrahepatic collateral arteries supplying the liver were readily evident. The collateral arteries originated from the SMA in 8 patients (72.7%), from the gastroduodenal artery in 2 patients (18.2%) and from the LGA in 1 patient (9.1%). The pancreaticoduodenal arcade, gastroduodenal artery, and the LGA were dilated on celiac axis angiograms. Angiography showed good opacification of the portal vein in this group. Transcatheter treatment was successfully performed in all patients except the one who had hemangiomas. Three of the patients underwent chemoembolization, and seven of the patients underwent hepatic arterial infusion chemotherapy via the collateral artery. Four weeks later, the transarterial procedure was performed for the patient whose collateral artery originated from the LGA. DSA showed complete occlusion of the LGA and the CHA, and the new collateral artery originated from the SMA. Chemoembolization was performed via the new collateral artery. All eleven patients (100%) had a normal outcome and did not develop any complications on follow-up (Table 1) (Figures 1 and 2).

## 4. Discussion

The main cause of extrahepatic collateral vessel development was believed to be hepatic artery occlusion by postinterventional dissection or embolization. Some authors advocate that hepatic artery interruption by repeated TACE or arterial dissection is the primary cause [14]. Kim et al. [15] demonstrated that only about 4% of patients with extrahepatic collateral vessel development had proximal hepatic artery occlusion, and most patients with a collateral supply had a widely patent hepatic artery. In this study, all the 11 patients with extrahepatic collateral vessel had proximal hepatic artery (CHA or PHA) occlusion related to interventional procedures.

There have been a few CT-, and CTA-based studies that evaluated the collateral routes related to HAO [12, 13]. Studies indicated that the collateral routes were immediately evident during HAO and that most collateral arteries originated from the inferior phrenic artery (IPA), the SMA, celiac axis, LGA, or the dorsal pancreatic artery [11–13, 15, 16]. Tohma et al. [13] studied the arterial collateral system of 13 patients with various cancers (6 hepatocellular carcinomas, 4 metastatic liver tumors from colorectal cancer, 2 distal common duct tumors, and 1 pancreatic cancer) at the hepatic hilum by using CT and CTA during temporary balloon occlusion of the right or left hepatic artery. They found that during temporary occlusion of the right or left hepatic artery, the communicating arcade between the right and left hepatic arteries was immediately evident in all patients. Their results indicated that the communicating arcade played an important role in the interlobar arterial collateral system. Takeuchi et al. [12] used CTA to evaluate the routes of potential extrahepatic arteries (supplying to the liver) of 23 patients with liver tumors before and after temporary balloon occlusion of the PHA. They found that during temporary balloon occlusion of the PHA, extrahepatic arterial supply was immediately evident in 22 of the 23 patients. The liver was supplied by the right IPA in 17 of 20 patients, by the left IPA in five of six, by the SMA in 8 of 16, by the celiac axis in 2 of 10, and by the LGA in 1 of 6. Murata et al. [11] reported 14 patients with liver tumors and HAO following reservoir placement. They found that the main collateral pathway of the feeding artery on angiography was the IPA in 7 patients (50%), the dorsal pancreatic artery in 4 patients (29%), and the anastomotic branch of the celiac axis in 1 patient (7%). The main collateral pathway could not be detected in 2 patients (14%). Kim et al. [15] demonstrated that extrahepatic collateral arteries commonly supply hepatocellular carcinomas if the tumors are large or peripherally located. They observed 2104 extrahepatic collateral vessels in 860 patients. The extrahepatic collateral vessels observed originated from the IPA, omental branch, adrenal artery, intercostal artery, cystic artery, internal mammary artery, renal or renal capsular artery, branch of the SMA, gastric artery, and lumbar artery. The right IPA was found to be the most common extrahepatic collateral vessel that supplies HCC [15, 17]. Kim et al. [15] indicated that when the tumor is located in liver segment S7 and is in contact with the right hemidiaphragm, selective angiography of the right IPA is mandatory. When the tumor is located in liver segments S2 or S3 and abuts the left hemidiaphragm, the possibility of a collateral blood supply from the left IPA should be kept in mind. In the present study, we found that the majority of collateral arteries originated from the SMA and that the most important and frequently encountered collateral vessels are the pancreaticoduodenal arcades. The collateral artery originated from the LGA in one patient, and angiography showed complete occlusion of the LGA; in addition, the new collateral arteries originated from the SMA four weeks later. Although we sometimes detected the right IPA as an extrahepatic collateral vessel that supplies HCC, we did not found it in this patient population. One reason maybe is that the tumors of this group were not located in liver segment S7 and were not in contact with the right hemidiaphragm. The other reason maybe is that the IPA was not evaluated in this group. Because selective angiography of individual collateral vessels is tedious and time consuming, it is essential to try to determine first whether collateral blood supply is present. The initial CT scan provides useful information, and CT signs of direct invasion into adjacent organs or extracapsular infiltration indicate the presence of extrahepatic collateral vessels [15]. In the future, we should review the preinterventional CT images to study the tumor scans and observe if there is the collateral blood supply and routinely do an angiographic workup including selective angiogram of the phrenic artery.

It has been shown that hepatic arterial infusion chemotherapy or chemoembolization is effective and significantly improves survival of patients with liver tumors [3–5]. However, HAO can disrupt transcatheter management of liver tumors. There have been a few reports about successful transcatheter management through the collateral arteries, which is a promising trend [11, 16–18]. Ikeda et al. [18] provide several tips on surmounting these difficulties in interventional radiology including transcatheter arterial chemoembolization for hepatocellular carcinoma and an implantable port system for hepatic arterial infusion chemotherapy to treat metastatic liver tumors. Chung et al. [17] reported that fifty patients with HCC underwent a total of 82 procedures of transcatheter oily chemoembolization therapy of the IPA, as well as of the hepatic artery. In 16 patients, additional extrahepatic collaterals were depicted and were also embolized in 10 patients. Their results indicated the efficacy and safety of transcatheter chemoembolization therapy via the extrahepatic collateral arteries in HCC. Murata et al. [11] performed transcatheter management in 14 patients for liver tumors after hepatic artery obstruction via the collateral pathway, and suggested that transcatheter treatment may be possible in patients with HAO. In the present study, transcatheter treatment was successfully performed via the collateral arteries in all patients except the one with hemangiomas.

Occlusion of the hepatic artery combined with concomitant portal vein occlusion leads to sharp segmental liver necrosis without formation of significant collaterals [19]. Vaidya et al. [20] studied adult orthotopic liver transplantation patients who underwent an angiography for suspected hepatic arterial abnormalities over an approximately 10-year period. They found that of the 129 angiographies, 24 (19.4%) were found to have collaterals on angiography, and eleven patients (41.7%) had complications related to the liver ischemia on follow-up. In this limited group, good portal venous inflow and collateral arterial flowto the liver prevented liver ischemia and infarction. There were no symptoms of liver ischemia or infarction in all the patients.

In summary, our results indicate that extrahepatic collateral arteries supplying the liver were readily evident in patients with HAO. They are important alternative routes for continuous transcatheter management of hepatic neoplasms following HAO. DSA is an effective method to detect collateral circulation related to HAO and may provide information to guide management decisions.

## Figures and Tables

**Table 1:** Baseline patient demographics and collateral artery characteristics.

Patient no.	Age (y)	Sex	Primary	Occlusion of hepatic artery	Collateral pathways	Therapeutic method
1	56	Male	Colon cancer	Common hepatic artery	Superior mesenteric artery	Hepatic arterial infusion chemotherapy
2	46	Male	Colon cancer	Common hepatic artery	Superior mesenteric artery	Hepatic arterial infusion chemotherapy
3	53	Male	Hepatocellular carcinoma	Common hepatic artery	Superior mesenteric artery	Transarterial chemoembolization
4	36	Male	Hepatocellular carcinoma	Common hepatic artery	Superior mesenteric artery	Hepatic arterial infusion chemotherapy
5	37	Male	Hepatocellular carcinoma	Common hepatic artery	Left gastric artery	Transarterial chemoembolization
6	59	Male	Hepatocellular carcinoma	Common hepatic artery	Superior mesenteric artery	Transarterial chemoembolization
7	29	Female	Hepatocellular carcinoma	Common hepatic artery	Superior mesenteric artery	Hepatic arterial infusion chemotherapy
8	60	Female	Hemangiomas	Common hepatic artery	Superior mesenteric artery	Resection
9	50	Female	Stomach cancer	Common hepatic artery	Superior mesenteric artery	Hepatic arterial infusion chemotherapy
10	70	Female	Hepatocellular carcinoma	Proper hepatic artery	Gastroduodenal artery	Hepatic arterial infusion chemotherapy
11	70	Male	Hepatocellular carcinoma	Proper hepatic artery	Gastroduodenal artery	Hepatic arterial infusion chemotherapy
